# Maraviroc, a chemokine receptor-5 antagonist, fails to demonstrate efficacy in the treatment of patients with rheumatoid arthritis in a randomized, double-blind placebo-controlled trial

**DOI:** 10.1186/ar3685

**Published:** 2012-01-17

**Authors:** Dona L Fleishaker, Juan A Garcia Meijide, Andriy Petrov, Michael David Kohen, Xin Wang, Sujatha Menon, Thomas C Stock, Charles A Mebus, James M Goodrich, Howard B Mayer, Bernhardt G Zeiher

**Affiliations:** 1 Pfizer Inc, 700 Chesterfield Parkway, St. Louis, MO, 63017, and Eastern Point Road, Groton, CT, 06340 USA; 2 Hospital Ntra. Sra. de la Esperanza, 2 Santiago de Compostela, a Coruña15705,Spain; 3 Crimean State Medical University, 69 Kyivska Street, Simferopol, 95017, Ukraine; 4 Avivoclin Clinical Services, 5111 S Ridgewood Avenue, Suite 301, Port Orange, FL, 32127,USA; 5 ViiV Healthcare, Five Moore Drive, Research Triangle Park, NC, 27709, USA; 6 EMD Serono Inc., One Technology Place, Rockland, MA, 02370, USA; 7 Astellas US LLC, Three Parkway North, Deerfield, IL, 60015, USA

## Abstract

**Introduction:**

The purpose of this study was to determine whether maraviroc, a human CC chemokine receptor 5 (CCR5) antagonist, is safe and effective in the treatment of active rheumatoid arthritis (RA) in patients on background methotrexate (MTX).

**Methods:**

This phase IIa study comprised two distinct components: an open-label safety study of the pharmacokinetics (PK) of MTX in the presence of maraviroc, and a randomized, double-blind, placebo-controlled, proof-of-concept (POC) component. In the PK component, patients were randomized 1:1 to receive maraviroc 150 or 300 mg twice daily (BID) for four weeks. In the POC component, patients were randomized 2:1 to receive maraviroc 300 mg BID or placebo for 12 weeks. Patients were not eligible for inclusion in both components.

**Results:**

Sixteen patients were treated in the safety/PK component. Maraviroc was well tolerated and there was no evidence of drug-drug interaction with MTX. One hundred ten patients were treated in the POC component. The study was terminated after the planned interim futility analysis due to lack of efficacy, at which time 59 patients (38 maraviroc; 21 placebo) had completed their week 12 visit. There was no significant difference in the number of ACR20 responders between the maraviroc (23.7%) and placebo (23.8%) groups (treatment difference -0.13%; 90% CI -20.45, 17.70; P = 0.504). The most common all-causality treatment-emergent adverse events in the maraviroc group were constipation (7.8%), nausea (5.2%), and fatigue (3.9%).

**Conclusions:**

Maraviroc was generally well tolerated over 12 weeks; however, selective antagonism of CCR5 with maraviroc 300 mg BID failed to improve signs and symptoms in patients with active RA on background MTX.

**Trial Registration:**

ClinicalTrials.gov: NCT00427934

## Introduction

Maraviroc is an orally active, noncompetitive, reversible antagonist of the human CC chemokine receptor 5 (CCR5), which is the primary chemokine receptor expressed by rheumatoid synovial T cells [1,2]. It has been approved for use in combination with other antiretroviral agents in treatment-experienced adult patients who have been infected with only CCR5-tropic HIV-1 and who have evidence of viral replication and HIV-1 strains resistant to multiple antiretroviral agents [3,4]. Studies have demonstrated reductions in viral load at maraviroc doses ranging from 150 to 600 mg twice daily (BID) (based on concomitant medications) [5]. In addition to having a role as a co-receptor for CCR5 HIV viral binding and cell entry, the CCR5 receptor has a role in the trafficking, localization, and differentiation of leukocytes [6,7]. In rheumatoid arthritis (RA), chemokine upregulation is associated with tissue and joint destruction and increased levels of CCR5 receptor ligands (Regulated on Activation, Normal T-cell Expressed, and Secreted [RANTES], macrophage inflammatory protein [MIP]-1α, and MIP-1β) in the synovial fluid [8-11]. It is hypothesized that, by preventing chemokine-induced CCR5 activation, cellular retention at sites of inflammation and activation of synovial fibroblasts and chondrocytes will be reduced, yielding both symptomatic relief and a reduction in joint destruction in patients with RA [1].

Preclinical work in a rhesus monkey collagen-induced arthritis model demonstrated suppression of C-reactive protein (CRP) and altered antibody response toward type II collagen with a CCR5-antagonist, SCH-X [12]. In another preclinical study, use of Met-RANTES, which blocks both CCR1 and CCR5, caused the amelioration of adjuvant-induced arthritis in Lewis rats [13]. Furthermore, there is evidence to suggest that the CCR5Δ32 mutation, which leads to reduced CCR5 expression at the cell surface, is associated with a protective effect in patients with RA [14-16]; however, this finding has not been consistent [17,18].

This study comprised two distinct components. The primary objectives of the first component were to evaluate the safety and tolerability of maraviroc 150 and 300 mg BID administered for 4 weeks to patients with active RA on stable background treatment with methotrexate (MTX), characterize the pharmacokinetics (PK), and investigate potential drug-drug interactions (DDIs) between maraviroc and MTX after 4 weeks of co-administration. The primary objective of the second component was to assess the safety and efficacy of maraviroc 300 mg BID (versus placebo) in patients with active RA on stable background treatment with MTX after 12 weeks of treatment.

## Materials and methods

This study was sponsored by Pfizer Inc (New York, NY, USA) and was conducted in 41 centers in nine countries (Australia, Germany, India, Italy, Mexico, Portugal, Spain, Ukraine, and the US). The final protocol, amendments, and informed consent documentation were reviewed and approved by the institutional review boards and the independent ethics committees at each of the investigational centers participating in the study. This study was conducted in compliance with the ethical principles originating in or derived from the Declaration of Helsinki and in compliance with the International Conference on Harmonization Good Clinical Practice guidelines. All patients provided informed consent prior to screening and enrollment. The trial is registered as http://ClinicalTrials.gov number NCT00427934.

### Patients

Eligible patients had to be at least 18 years old, had to have an active-RA diagnosis based on the American College of Rheumatology (ACR) 1987 revised criteria, and had to meet the ACR 1991 Revised Criteria for Global Functional Status in RA (class I, II, or III). Patients had to be receiving MTX therapy for at least 12 weeks prior to study entry at a stable dose for at least 4 weeks prior to study entry which remained unchanged throughout the 12 week treatment period. MTX dosage had to be at least 10 mg/week and not more than 25 mg/week (oral or parenteral) unless documented intolerance required a lower dose. In the safety/PK component, there were no disease activity requirements. However, in the proof-of-concept (POC) component, a minimum disease activity requirement of at least six tender/painful joints on motion (28-joint count), at least six swollen joints (28-joint count), a CRP of at least 0.7 mg/dL, or an erythrocyte sedimentation rate of at least 28 mm/hour was necessary for inclusion.

Patients were excluded if they were receiving or unable to washout other medications that could interfere with disease activity assessments. Such medications would include the following: auranofin, injectable gold, sulfasalazine, d penicillamine, azathioprine, cyclosporine, high-dose corticosteroids (> 10 mg/day prednisone or the equivalent), anakrinra, etanercept, herbal supplements that included fish oil, or leflunomide (within 4 weeks); infliximab, adalimumab, or any experimental RA therapy (within 8 weeks); abatacept (within 3 months); or rituximab (within 12 months). Other exclusion criteria included the following: a history of chronic life-threatening infection; severe, progressive, and/or uncontrolled renal, hepatic, hematological, gastrointestinal, endocrine, pulmonary, cardiac, or neurologic disease; tuberculosis without treatment and/or positive tuberculin reaction without known Bacilli Calmette-Guérin vaccination; positive homozygous CCR5Δ32 mutation; history or evidence of postural hypotension; New York Heart Association class III-IV congestive heart failure requiring treatment; mean corrected QT (QTc) interval of greater than 450 ms; evidence of any current active infection; or history of cancer and in remission for less than 3 years.

### Study design

**Figure 1:**
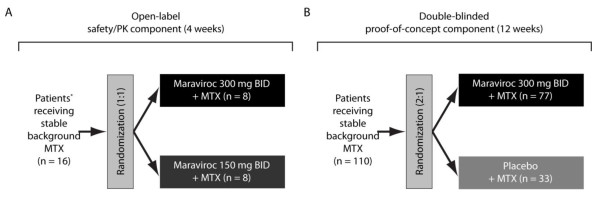
**Study design**. **(A) **Open-label safety/PK component (4 weeks). **(B) **Double-blinded proof-of-concept component (12 weeks). *Patients without any disease activity requirements. BID, twice daily; MTX, methotrexate; PK, pharmacokinetics.

This phase IIa study investigated oral maraviroc in patients who had RA and who were receiving background MTX. The study comprised two components: an open-label safety/PK component and a randomized, double-blind, placebo-controlled POC component (Figure 1).

### Safety/pharmacokinetics component

This component comprised a 4-week, open-label study conducted only in the US. Two dose levels of oral maraviroc (150 mg and 300 mg BID) were investigated in patients who had no disease activity requirements and who were on stable doses of background MTX (Figure 1A). This component of the study was conducted to confirm the safety and lack of clinically significant DDIs when maraviroc was co-administered with MTX. Patients enrolled in this portion of the study were not eligible for inclusion in the POC component of the study, and replacements could be recruited if patients were discontinued from the study for reasons other than safety.

Blood samples were obtained at screening visits and were used to assess each patient's steady-state MTX PK parameters. Additional blood samples were collected at week 1 in order to assess steady-state MTX PK parameters in the presence of maraviroc. PK analyses for potential DDIs, coupled with weekly safety monitoring, were used by an internal risk management committee to select a dose for the POC component of the study. If the safety and PK profiles of the 150- and 300-mg doses were similar and there was no evidence of DDI with MTX, the 300-mg dose would be selected for the 12-week POC component.

### Proof-of-concept component

Eligible patients on stable background doses of MTX were randomly assigned 2:1 to either oral maraviroc 300 mg BID or placebo BID for 12 weeks (Figure 1B). Safety and efficacy assessments were performed at baseline and at weeks 1, 2, 4, 8, and 12, and a follow-up visit took place at week 16. An interim futility analysis was planned after approximately 57 patients had completed their week 12 visit and was performed by an internal data-monitoring committee.

### Treatments

All doses of maraviroc were self-administered and taken at approximately 12 hour intervals on an empty stomach, specifically 1 hour before a meal or 4 hours after the last meal. Both maraviroc and placebo treatments were added to ongoing stable background MTX therapy.

### Concomitant treatments

All medications taken 28 days prior to the first dose of study treatment had to be recorded. Patients were permitted to continue on their stable background RA therapy, which could have included nonsteroidal anti-inflammatory drugs and cyclooxygenase-2 inhibitors, low-dose oral corticosteroids (not more than 10 mg of prednisone per day or the equivalent), and opioid analgesics (not more than 30 mg of oral morphine per day or the equivalent). Concomitant use of not more than 325 mg/day of aspirin was acceptable if taken for nonarthritic reasons. In addition, concomitant use of folic acid was encouraged for patients receiving MTX. Use of moderate to strong CYP3A4 inhibitors/inducers and grapefruit products was prohibited during the study. In the POC component, patients on stable doses of antimalarials (chloroquine and hydroxychloroquine) for at least 60 days were allowed to continue these medications.

### Rescue medications

Acetaminophen (paracetamol) (not more than 2.6 g/day for not more than four consecutive days) was allowed as a rescue medication. If a patient was already taking stable background doses of acetaminophen, the dose could be increased up to 2.6 g/day for up to four consecutive days for rescue purposes. Rescue medication was not permitted within 24 hours of a study visit. Patients who required rescue medication for more than four consecutive days were discontinued from the study for lack of efficacy.

### Evaluations

#### Safety/pharmacokinetics component

In addition to the general safety evaluations outlined below, maraviroc and MTX PK parameters were calculated for each patient for each treatment by using noncompartmental analysis of concentration-time data. Peak plasma concentration (C_max_) and time to C_max _(T_max_) were determined by inspection of the individual concentration-time profiles. The area under the plasma concentration-time profile from time 0 to 4 hours postdose (AUC_0-4_) was determined by the linear/log linear trapezoidal method.

#### Proof-of-concept component

The primary efficacy variable was ACR20 (American College of Rheumatology 20% improvement criteria) response rate at week 12. Other efficacy variables included the following: ACR50, ACR70, patient's assessment of arthritis pain, patient's global assessment of arthritis, physician's global assessment of arthritis, health assessment questionnaire-disability index, CRP variable of the four-variable disease activity score using 28 joint counts (DAS28-4 [CRP]) and the two component scores of the Short Form-36 (physical and mental component summary) health questionnaire. Patients had to remain 'on study, off drug' up to 28 days after the last dose, at which time the week 16 visit procedures had to be completed.

### Safety evaluations

Safety was evaluated by monitoring adverse events (AEs), clinical laboratory evaluations, vital signs, and 12-lead electrocardiograms (ECGs) and by conducting physical examinations. All treatment-emergent AEs were summarized by body system and preferred term within each treatment group by using the Medical Dictionary for Regulatory Activities (MedDRA version 11.1; Maintenance and Support Services Organization).

### Statistical analyses

In the POC component, it was anticipated that 30% of patients assigned to receive placebo and 55% of patients assigned to receive maraviroc 300 mg BID would achieve an ACR20 response at week 12. Sample sizes of 38 patients for the placebo group and 76 patients for the maraviroc group were considered sufficient to detect an absolute difference of 25% in ACR20 response with 81% power and a type I error of 0.05 in a one-sided test, taking the planned futility interim analysis into account. The primary efficacy endpoint in the POC component was performed on the full analysis set (FAS), which was defined as all randomly assigned patients who received at least one dose of study drug. ACR20 response rate at week 12 was also analyzed for the interim analysis set (IAS) and observed cases. Categorical variables (ACR20, ACR50, and ACR70) were analyzed by the chi-squared test unless the normal approximation to the binomial distribution was not appropriate. If this was the case, the Barnard exact test was used. Analysis of covariance models, with treatment and region as fixed effect and baseline as covariate, was performed for the components of ACR response and DAS28 endpoints.

## Results

### Patient disposition

**Figure 2:**
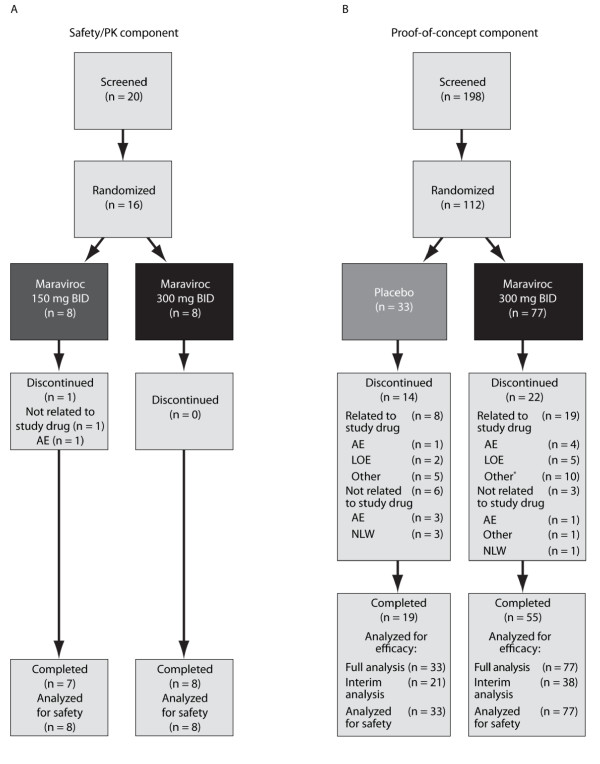
**Patient disposition**. **(A) **Safety/PK component. **(B) **Proof-of-concept component. *Discontinued because the study was terminated by the sponsor. AE, adverse event; BID, twice daily; LOE, lack of efficacy; NLW, no longer willing (to participate in study).

Sixteen patients were randomly assigned and treated in the safety/PK component: eight patients received maraviroc 150 mg BID, and eight patients received maraviroc 300 mg BID in addition to their stable background MTX treatment (Figure 2A). One patient in the maraviroc 150 mg BID group discontinued treatment because of an AE of worsening RA (moderate).

Of the 112 patients assigned to the POC component, 110 patients were treated: 77 with maraviroc 300 mg BID and 33 with placebo. Random assignment was allowed to continue during the interim analysis; hence, more patients were included in the final data analysis than in the interim. Of the 198 patients screened for the CCR5Δ32 mutation in the POC component, only one patient presented with the homozygous mutation and was subsequently excluded from the study. In total, 74 (66.1%) patients completed the POC component of the study, and 14 (42.4%) and 22 (28.6%) patients withdrew from the placebo and maraviroc treatment groups, respectively (Figure 2B). The numbers of patients who withdrew for reasons related to study treatment were eight (24.2%) and 19 (24.7%) in the placebo and maraviroc groups, respectively.

### Baseline demographics and characteristics

Baseline patient demographics and characteristics for both the safety/PK and POC components are shown in Table 1. The mean ages of patients in the safety/PK component were 58.5 years (maraviroc 150 mg BID) and 56.6 years (maraviroc 300 mg BID); for the POC component, the mean ages were 53.6 and 53.4 years in the maraviroc 300 mg BID and placebo groups, respectively. While the percentages of males and females in the safety/PK component were similar, 92.2% of patients in the maraviroc 300 mg BID group and 66.7% in the placebo group in the POC component were female. The mean MTX doses were 14.27 mg/week (range of 7.5 to 30 mg/week) in the POC component and 16.41 mg/week (range of 10 to 22.5 mg/week) in the PK component. The mean durations from first diagnosis of RA at baseline were similar in the maraviroc 150 and 300 mg BID groups in the safety/PK component (11.0 and 11.9 years, respectively) and in the POC component: 7.8 years (placebo) and 7.9 years (maraviroc 300 mg BID). Patients in the POC component had moderate to severe active RA with mean DAS28-4 (CRP) values of 6.0 and 5.8 in the placebo and maraviroc treatment groups, respectively. Components of the DAS were also similar between placebo and maraviroc 300 mg BID at baseline in the POC component (Table 1).

**Table 1:** Baseline patient demographics and characteristics

	Safety/PK component		POC component		
	**Maraviroc 150 mg BID (*n *= 8)**	**Maraviroc 300 mg BID (*n *= 8)**	**Placebo** **(*n *= 33)**	**Maraviroc 300 mg BID (*n *= 77)**	***P *value^a^**

Female, number (percentage)	4 (50.0)	5 (62.5)	22 (66.7)	71 (92.2)	0.0014
Age in years					
Mean (SD)	58.5 (7.8)	56.6 (5.9)	53.4 (11.1)	53.6 (12.1)	0.9274
Range	44-67	46-63	34-76	20-81	-
Race, number (percentage)					-
White	8 (100.0)	8 (100.0)	21 (63.6)	51 (66.2)	
Black	0	0	0	3 (3.9)	
Asian	0	0	5 (15.2)	7 (9.1)	
Other	0	0	7 (21.2)	16 (20.8)	
Weight in kg, mean (SD)	90.9 (30.4)	88.9 (13.9)	72.7 (16.9)	70.5 (15.4)	0.5173
Height in cm, mean (SD)	164.5 (11.1)	167.4 (4.8)	162.0 (9.7)	159.8 (7.6)	0.2057
BMI in kg/m^2^, mean (SD)	33.3 (10.2)	31.6 (4.0)	27.6 (5.5)	27.5 (5.4)	0.9670
Duration from first diagnosis^b^					0.9817
Mean in years	11.0	11.9	7.8	7.9	
Range in years	3.0-22.3	2.5-29.0	0.6-32.0	0.4-40.0	
ACR components, mean (SD)^c^					
Tender/painful joint count	-	-	17.5 (6.6)	16.4 (7.3)	0.3565
Swollen joint count	-	-	12.8 (5.9)	11.5 (4.6)	0.2062
Patient's assessment of arthritis pain	-	-	57.9 (26.0)	59.3 (20.6)	0.6375
Patient's global assessment of arthritis	-	-	61.8 (26.1)	61.3 (21.2)	0.8932
Physician's global assessment of arthritis	-	-	3.5 (0.7)	3.5 (0.7)	0.7926
HAQ-DI	-	-	1.7 (0.7)	1.7 (0.6)	0.8405
CRP	-	-	17.4 (19.0)	14.3 (13.2)	0.6946
DAS28-4 (CRP)^c^	-	-	6.0 (0.9)	5.8 (0.8)	0.4554

^a^Two-sample *t *test comparison for proof-of-concept (POC) component demographics. ^b^To day 1 of study. ^c^American College of Rheumatology (ACR) components and DAS28-4 (CRP) were not assessed at baseline for the safety/pharmacokinetic (PK) component. BID, twice daily; BMI, body mass index; CRP, C-reactive protein; DAS28-4 (CRP), disease activity score, 28-joint count, using C-reactive protein; HAQ-DI, health assessment questionnaire-disease index; SD, standard deviation.

### Efficacy

An interim analysis was performed when approximately 50% of the total planned patients completed the study. Fifty-nine patients (38 maraviroc 300 mg BID and 21 placebo) completed the week 12 interim analysis visit. There was no significant difference in the percentage of ACR20 responders at week 12 in the maraviroc (23.7%) and placebo (23.8%) groups: difference from placebo of -0.13; 90% confidence interval (CI) of -20.45 to 17.70; *P *= 0.504 (IAS). The O'Brien-Fleming futility boundary [19] (4.5% in observed difference of maraviroc from placebo) was crossed for the primary analysis of ACR20 response, and the study was therefore terminated. Random assignment of new patients and study drug administration for all previously randomly assigned patients was discontinued.

**Figure 3:**
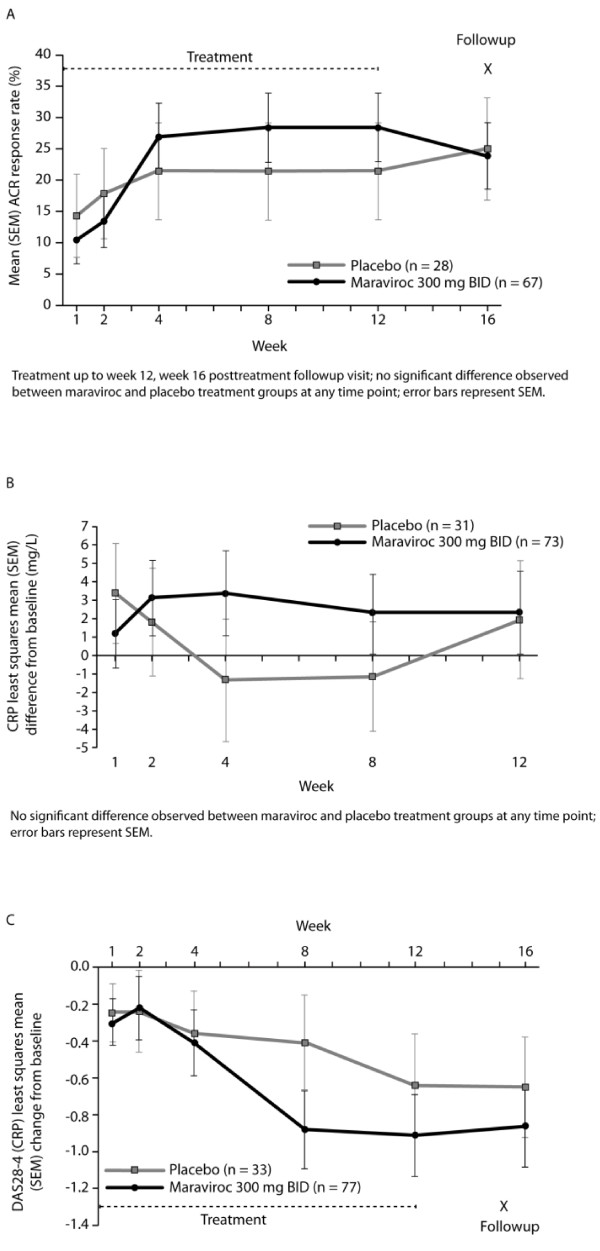
**POC Component: Key Efficacy Endpoints **(A) **Effect of maraviroc on ACR20 response rate over time**. **(B) **Least squares mean change from baseline in CRP over time. **(C) **Least squares mean change from baseline in DAS28-4 (CRP) over time; all figures used data from the full analysis set (FAS) with last observation carried forward(LOCF). ACR20, American College of Rheumatology 20% improvement criteria; BID, twice daily; CRP, C-reactive protein; DAS, disease activity score; DAS28-4 (CRP), disease activity score, 28-joint count, using C-reactive protein; FAS, full analysis set; LOCF, last observation carried forward; SEM, standard error of the mean.

At the final analysis, no significant difference was seen for the ACR20 response at week 12 (FAS) between maraviroc 300 mg BID (28.4%) and placebo (21.4%): difference from placebo of 9.09; 90% CI of -6.16 to 21.83; *P *= 0.155. There was no significant difference between maraviroc 300 mg BID and placebo groups for ACR20 response at any time point (Figure 3A).

Sensitivity analyses also failed to demonstrate a significant difference. Observed case analysis showed no significant difference for ACR20 response at week 12 between maraviroc 300 mg BID (30.0%) and placebo (19.1%): difference from placebo of 10.95; 90% CI of -9.50 to 29.05; *P *= 0.225.

A small percentage of patients exhibited ACR50 and ACR70 responses throughout the study period. The numbers of patients with an ACR50 response at at least one study visit were 11 (15.3%) and four (12.9%) in the maraviroc and placebo groups, respectively. The numbers of patients with an ACR70 response at at least one study visit were two (2.8%) and three (9.7%) in the maraviroc and placebo groups, respectively.

At week 12, CRP appeared to increase from baseline in the maraviroc group and decrease from baseline in the placebo group; however, no significant difference between the groups was observed at any time point over 12 weeks (Figure 3B). At week 12, the least squares mean changes from baseline in CRP were 2.35 (maraviroc 300 mg BID) and 1.93 (placebo) (*P *= 0.912). DAS28-4 (CRP) decreased from baseline in both treatment groups over the 12 week study period (Figure 3C); at week 12, the least squares mean changes from baseline were -0.91 (maraviroc 300 mg BID) and -0.64 (placebo) (*P *= 0.339). There were no significant differences at any time point in DAS28-4 (CRP).

Changes from baseline in other efficacy variables at week 12 are summarized in Table 2. For components of the ACR20 response, patients in both treatment groups demonstrated changes in their assessment of arthritis pain and global assessment of arthritis but not at clinically significant levels.

**Table 2:** Summary of least squares mean (SEM) change from baseline in secondary efficacy endpoints at week 12 (proof-of-concept component; FAS, LOCF)

	Placebo	Maraviroc 300 mg BID	*P *value
ACR components			
Tender/painful joint count	-3.41 (1.19) [*n *= 33]	-4.89 (0.84) [*n *= 77]	0.294
Swollen joint count	-3.43 (0.95) [*n *= 33]	-3.48 (0.66) [*n *= 77]	0.966
Patient's assessment of arthritis pain	-6.09 (4.06) [*n *= 33]	-8.30 (2.85) [*n *= 77]	0.644
Patient's global assessment of arthritis	-6.78 (4.06) [*n *= 33]	-8.55 (2.88) [*n *= 76]	0.712
Physician's global assessment of arthritis	-0.36 (0.15) [*n *= 32]	-0.49 (0.11) [*n *= 75]	0.490
HAQ-DI	-0.06 (0.13) [*n *= 20]	-0.18 (0.08) [*n *= 55]	0.396
SF-36 version 2 (acute)			
Physical component summary	4.81 (1.65) [*n *= 19]	3.14 (1.04) [*n *= 53]	0.344
Mental component summary	0.61 (2.25) [*n *= 19]	1.17 (1.41) [*n *= 53]	0.818

ACR, American College of Rheumatology; BID, twice daily; FAS, full analysis set; HAQ-DI, health assessment questionnaire-disability index; LOCF, last observation carried forward; SEM, standard error of the mean; SF-36, Short Form-36 Health Survey.

### Safety

Maraviroc was generally well tolerated when given in combination with stable background doses of MTX in both the safety/PK and POC components of this study.

### Safety/pharmacokinetics component

A total of four (50%) patients in each treatment group experienced at least one AE. In the maraviroc 150 mg BID group, two patients reported AEs that were considered by the study investigator to be treatment-related (nausea, fatigue, arthralgia, back pain, and muscle spasms). In the maraviroc 300 mg BID group, one patient reported treatment-related AEs: abnormal feces and dry mouth. The AE profile was very similar in both dosing groups, and no serious or severe AEs, temporary discontinuations, dose reductions due to AEs, or deaths were reported. Infections and infestations were reported in two (25%) patients in the maraviroc 300 mg BID group, but none was considered treatment-related. Vital signs and ECG parameters remained stable during the study period, and no clinically significant changes were noted. Laboratory abnormalities were reported in six (75%) and seven (88%) patients in the maraviroc 150 mg BID and 300 mg BID groups, respectively.

### Proof-of-concept component

Treatment-emergent and treatment-related AEs (reported by at least two patients in either treatment arm) are shown in Table 3. The majority of AEs were mild to moderate in severity, and a total of 60 (55%) patients reported at least one treatment-emergent AE. The most common all-causality treatment-emergent AEs in the maraviroc group were constipation (7.8%), nausea (5.2%), worsening RA (3.9%), fatigue (3.9%), upper respiratory tract infection (3.9%), and respiratory tract infection (2.6%). In the placebo group, the most common all-causality treatment-emergent AEs were worsening RA (24.2%), diarrhea (6.1%), peripheral edema (6.1%), and influenza (6.1%). No serious AEs were reported during the study. In the placebo group, one case of neutropenia, visual impairment, and ulcer and two cases of RA were classed as severe. In the maraviroc group, there was one case of humerus fracture and one of RA, and both were considered to be severe. Infections and infestations were reported in 10 (13.0%) patients in the maraviroc 300 mg BID group and five (15.2%) patients in the placebo group; of these, only one instance in the placebo group was considered treatment-related. The most common infections and infestations were upper respiratory tract infection (three patients in the maraviroc 300 mg BID group), respiratory tract infection (two patients in the maraviroc 300 mg BID group), and influenza (two patients in the placebo group).

**Table 3:** Summary of treatment-emergent and treatment-related adverse events by preferred term reported by at least two patients in either treatment group (proof-of-concept component)

Adverse events, number (percentage)	Placebo		Maraviroc 300 mg BID	
	**Treatment-emergent** **(*n *= 33)**	**Treatment-related** **(*n *= 33)**	**Treatment-emergent** **(*n *= 77)**	**Treatment-related** **(*n *= 77)**

Worsening rheumatoid arthritis	8 (24.2)	1 (3.0)	3 (3.9)	0
Constipation	0	0	6 (7.8)	4 (5.2)
Nausea	0	0	4 (5.2)	3 (3.9)
Chills	1 (3.0)	0	2 (2.6)	2 (2.6)
Dizziness	1 (3.0)	1 (3.0)	2 (2.6)	1 (1.3)
Fatigue	0	0	3 (3.9)	1 (1.3)
Edema peripheral	2 (6.1)	2 (6.1)	0	0
Headache	1 (3.0)	0	2 (2.6)	1 (1.3)
Diarrhea	2 (6.1)	0	1 (1.3)	1 (1.3)
Upper respiratory tract infection	0	0	3 (3.9)	0
Orthostatic hypotension	0	0	2 (2.6)	1 (1.3)
Dyspepsia	0	0	2 (2.6)	1 (1.3)
Pyrexia	0	0	2 (2.6)	1 (1.3)
Respiratory tract infection	0	0	2 (2.6)	0
Influenza	2 (6.1)	0	0	0

BID, twice daily.

There were no clinically significant changes in vital signs or ECG measurements. Laboratory abnormalities were reported in 43 (64%) and 17 (61%) patients in the maraviroc 300 mg BID and placebo groups, respectively, but were not clinically significant. There was a slight increase in the cluster of differentiation-8 (CD8) counts (> 1.1 × upper limit of normal range, or ULN) of the maraviroc-treated patients (4%) in comparison with placebo (0%); however, CD4 counts (> 1.1 × ULN) were similar in both treatment groups. Maraviroc did not appear to impact hepatic function: there were no grade 2, 3, or 4 shifts in either treatment group for liver transaminases; the only significant alanine transaminase (ALT) elevation (> 3.0 × ULN) occurred in the placebo group.

### Pharmacokinetics and pharmacogenomics

Concomitant administration of MTX and maraviroc (150 or 300 mg BID) did not result in altered MTX exposure in patients with RA. After oral administration of maraviroc, absorption was rapid, and a median T_max _ranged from 2 to 2.5 hours in both doses studied. Increases in both AUC and C_max _were approximately proportional to the dose increases. Between-patient variability were 48% (150 mg BID) and 74% (300 mg BID) for AUC_0-4 _and 50% (150 mg BID) and 67% (300 mg BID) for C_max_. MTX concentration profiles at screening and 1 week after initiation of maraviroc were similar in both treatment groups. The changes in MTX exposure (based on the ratio of AUCs) were 0.96 and 1.18 after concomitant administration of maraviroc 150 mg BID and 300 mg BID, respectively. This magnitude of change in systemic exposure was not considered to be clinically relevant.

## Discussion

The objectives of this study were to evaluate the safety/PK profile of maraviroc in patients with RA and to investigate whether maraviroc-induced CCR5 antagonism reduces disease activity in these patients. The study demographic characteristics were generally well balanced, with the exception of the larger percentage of females in the maraviroc-treated subjects in the POC component. While this could have had some effect on treatment outcome, it is unlikely that the gender imbalance led to the lack of treatment effect observed in this trial.

The study stopped at a planned interim analysis because of futility. These findings were confirmed by the final analysis, demonstrating that there was no significant effect on ACR responder rates, CRP, or DAS. These results support a recent POC study that also demonstrated a lack of supporting evidence for the use of a CCR5 blockade as a therapeutic target in patients with active RA [20].

Given the well-documented action of maraviroc on CCR5 (a receptor that may play an important role in RA) [1], it is somewhat surprising that no significant improvements in the primary or secondary efficacy variables were observed in this study, as the maraviroc dose has demonstrated greater than 90% receptor occupancy [21].

One possible explanation may be that selective antagonism of CCR5 is insufficient to significantly improve all of the measures assessed in this study; blocking a number of chemokines may be necessary. This was postulated by van Kuijk and colleagues [20], who noted that the blockade of CCR1 or CCR2 alone yielded no significant improvement in RA. Alternatively, CCR5 may not be a relevant mechanism in the progression of RA. Another consideration is the timing of this intervention. Patients enrolled in the POC component of this study had a mean disease duration of nearly 8 years and had persistent disease activity despite treatment with MTX; this may be too late in the course of RA. Part of the rationale for studying maraviroc in patients with RA is the apparent protective effect conferred by the Δ32 mutation of CCR5 [14-16]. However, it may be that therapeutic intervention is required before the disease is clinically evident in order to be effective. It is also possible that the physiologic effects of this genetic deletion are different from those elicited by CCR5 antagonism with an oral small-molecule inhibitor after the onset of the disease. Furthermore, as corticosteroids are thought to affect leukocyte trafficking [22,23], it may be useful to compare efficacy profiles between those patients treated with concomitant corticosteroids and those who are not. In the present study, approximately 51% of patients received a corticosteroid during the trial; however, the numbers were too small to carry out a statistical comparison.

Results from the safety/PK portion of this study demonstrated that maraviroc 150 and 300 mg BID were well tolerated over 4 weeks and not associated with a DDI with MTX. In addition, there was no evidence to suggest that maraviroc meaningfully alters the PK disposition of MTX in patients with RA. The C_max _of maraviroc observed in this study was comparable to that achieved in asymptomatic patients with HIV but lower than that in healthy volunteers; T_max _values were comparable to those in healthy volunteers [24]. As the safety and PK profiles of maraviroc 150 and 300 mg were similar and there was no evidence of a DDI with MTX, the 300-mg dose was selected for administration in the 12-week POC component.

Results from the POC component of the study indicate that combined maraviroc 300 mg BID and MTX treatment was generally well tolerated and that the AE profile was very similar to that of both healthy volunteers and treatment-experienced patients infected with CCR5-tropic HIV in the early-phase I/II studies in the HIV indication [25,26], pivotal phase III studies [27], and postmarketing surveillance (data on file). Maraviroc was well tolerated in patients with RA, and there were no safety concerns in this study. Several early studies highlighted concerns about the possible class-specific, long-term hepatotoxic side effects of CCR5 antagonists [5,28], and the prescribing information for maraviroc contains a black-box warning for hepatotoxicity [24]; however, these warnings initially stemmed from studies using the chemokine antagonist, aplaviroc, in which several cases of severe liver toxicity emerged in patients receiving the drug, and trials were halted [29,30]. However, Nichols and colleagues [30] suggested that the hepatotoxicity of aplaviroc could be idiosyncratic rather than CCR5-mediated, as it was observed in animal models in which aplaviroc does not bind to CCR5. In the present study, even with the co-administration of MTX, there were no commonly reported AEs associated with hepatic function. Similarly, there were no discontinuations due to liver function in the maraviroc group, supporting other investigations of maraviroc in the HIV indication [31]. In addition, known systemic allergic symptoms that may precede idiosyncratic hepatotoxicity of allergic origin (such as elevation of eosinophils and incidence of an itchy rash) [26] were low (eosinophils) or nonexistent (rash).

## Conclusions

In summary, maraviroc 300 mg BID demonstrated an acceptable safety profile and was well tolerated but was not clinically efficacious in patients who had active RA and who were on background MTX.

## Abbreviations

ACR: American College of Rheumatology; ACR20: American College of Rheumatology 20% improvement criteria; ACR50: American College of Rheumatology 50% improvement criteria; ACR70: American College of Rheumatology 70% improvement criteria; AE: adverse event; AUC: area under the plasma concentration-time profile; AUC_0-4_: area under the plasma concentration-time profile from time 0 to 4 hours postdose; BID: twice daily; CCR1: CC chemokine receptor type 1; CCR5: human CC chemokine receptor 5; CI: confidence interval; C_max_: peak plasma concentration; CRP: C-reactive protein; DAS: disease activity score; DAS28-4 (CRP):disease activity score: 28-joint count: using C-reactive protein; DDI: drug-drug interaction; ECG: electrocardiogram; FAS: full analysis set; IAS: interim analysis set; MIP: macrophage inflammatory protein; MTX: methotrexate; PK: pharmacokinetics; POC: proof-of-concept; RA: rheumatoid arthritis; RANTES: Regulated upon Activation: Normal T-cell Expressed: and Secreted; T_max_: time to peak plasma concentration; ULN: upper limit of normal range.

## Competing interests

This study was funded by Pfizer Inc. DLF, XW, SM, TCS, and CAM are employees of Pfizer Inc. JMG, HBM, and BGZ were employed by Pfizer Inc at the time of the study. JAGM and MDK received clinical grant payments for the subjects they enrolled in the study. AP received clinical grant payments for the subjects he enrolled in the study and received grant support from Pfizer Inc.

## Authors' contributions

DLF was responsible for the study design, analysis and interpretation of the data, and manuscript preparation. JAGM, AP, and MDK were responsible for acquisition of the data, analysis and interpretation of the data, and manuscript preparation. XW was responsible for the study design, analysis and interpretation of data, statistical analysis, and manuscript presentation. SM was responsible for study design, analysis and interpretation of pharmacokinetic data, and manuscript preparation. TCS was responsible for medical oversight, analysis and interpretation of data, and manuscript preparation. CAM, JMG, and HBM were responsible for analysis and interpretation of the data and manuscript preparation. BGZ was responsible for study design and medical oversight while employed at Pfizer Inc, analysis and interpretation of data, and manuscript preparation. All authors read and approved the final manuscript.

## References

[B1] Gomez-Reino JJ Pablos JL Carreira PE Santiago B Serrano L Vicario JL Balsa A Figueroa M De Juan MD Association of rheumatoid arthritis with a functional chemokine receptor, CCR5 Arthritis Rheum 1999 42 989 992 10.1002/1529-0131(199905)42:5<989::AID-ANR18>3.0.CO;2-U 10323455

[B2] Abel S Back DJ Vourvahis M Maraviroc: pharmacokinetics and drug interactions Antivir Ther 2009 14 607 618 19704163

[B3] Fadel H Temesgen Z Maraviroc Drugs Today (Barc) 2007 43 749 758 10.1358/dot.2007.43.11.1131763 18174962

[B4] Meanwell NA Kadow JF Maraviroc, a chemokine CCR5 receptor antagonist for the treatment of HIV infection and AIDS Curr Opin Investig Drugs 2007 8 669 681 17668369

[B5] Lieberman-Blum SS Fung HB Bandres JC Maraviroc: a CCR5-receptor antagonist for the treatment of HIV-1 infection Clin Ther 2008 30 1228 1250 10.1016/S0149-2918(08)80048-3 18691983

[B6] Castellino F Huang AY tan-Bonnet G Stoll S Scheinecker C Germain RN Chemokines enhance immunity by guiding naive CD8+ T cells to sites of CD4+ T cell-dendritic cell interaction Nature 2006 440 890 895 10.1038/nature04651 16612374

[B7] Luther SA Cyster JG Chemokines as regulators of T cell differentiation Nat Immunol 2001 2 102 107 10.1038/84205 11175801

[B8] Haringman JJ Smeets TJM Reinders-Blankert P Tak PP Chemokine and chemokine receptor expression in paired peripheral blood mononuclear cells and synovial tissue of patients with rheumatoid arthritis, osteoarthritis, and reactive arthritis Ann Rheum Dis 2006 65 294 300 10.1136/ard.2005.037176 16107514 PMC1798063

[B9] Mack M Bruhl H Gruber R Jaeger C Cihak J Eiter V Plachy J Stangassinger M Uhlig K Schattenkirchner M Schlondorff D Predominance of mononuclear cells expressing the chemokine receptor CCR5 in synovial effusions of patients with different forms of arthritis Arthritis Rheum 1999 42 981 988 10.1002/1529-0131(199905)42:5<981::AID-ANR17>3.0.CO;2-4 10323454

[B10] Patel DD Zachariah JP Whichard LP CXCR3 and CCR5 ligands in rheumatoid arthritis synovium Clin Immun 2000 98 39 45 10.1006/clim.2000.4957 11141325

[B11] Ruth JH Rottman JB Katschke KJ Qin S Wu L LaRossa G Ponath P Pope RM Koch AE Selective lymphocyte chemokine receptor expression in the rheumatoid joint Arthritis Rheum 2001 44 2750 2760 10.1002/1529-0131(200112)44:12<2750::AID-ART462>3.0.CO;2-C 11762935

[B12] Vierboom MPM Zavodny PJ Chou C Tagat JR Pugliese-Sivo C Strizki J Steensma RW McCombie SW elebi-Paul L Remarque E Jonker M Narula SK Hart B Inhibition of the development of collagen-induced arthritis in rhesus monkeys by a small molecular weight antagonist of CCR5 Arthritis Rheum 2005 52 627 636 10.1002/art.20850 15693002

[B13] Shahrara S Proudfoot AE Woods JM Ruth JH Amin MA Park CC Haas CS Pope RM Haines GK Zha YY Koch AE Amelioration of Rat Adjuvant-Induced Arthritis by Met RANTES Arthritis Rheum 2005 52 1907 1919 10.1002/art.21033 15934086 PMC1282452

[B14] Pokorny V McQueen F Yeoman S Merriman M Merriman A Harrison A Highton J McLean L Evidence for negative association of the chemokine receptor CCR5 d32 polymorphism with rheumatoid arthritis Ann Rheum Dis 2005 64 487 490 15331395 10.1136/ard.2004.023333 PMC1755415

[B15] Prahalad S Negative association between the chemokine receptor CCR5-Delta 32 polymorphism and rheumatoid arthritis: a meta-analysis Genes Immun 2006 7 264 268 10.1038/sj.gene.6364298 16541097 PMC3104293

[B16] Rossol M Pierer M Arnold S KeyBer G Burkhardt H Baerwald C Wagner U Negative association of the chemokine receptor CCR5 d32 polymorphism with systemic inflammatory response, extra-articular symptoms and joint erosion in rheumatoid arthritis Arthritis Res Ther 2009 11 R91 10.1186/ar2733 19538721 PMC2714147

[B17] Kohem CL Brenol JC Xavier RM Bredemeier M Brenol CV Dedavid e Silva TL de Castilhos Mello A Canedo AD Neves AG Chies JAB The chemokine receptor CCR5 genetic polymorphism and expression in rheumatoid arthritis patients Scand J Rheumatol 2007 36 359 364 10.1080/03009740701393999 17963165

[B18] Zapico I Coto E Rodriguez A Torre JC Alvarez V CCR5 (chemokine receptor-5) DNA-polymorphism influences the severity of rheumatoid arthritis Genes Immun 2000 1 288 289 10.1038/sj.gene.6363673 11196706

[B19] Lan KK Lachin JM Bautista O Over-ruling a group sequential boundary--a stopping rule versus a guideline Stat Med 2003 22 3347 3355 10.1002/sim.1636 14566919

[B20] van Kuijk AW Vergunst CE Gerlag DM Bresnihan B Gomez-Reino JJ Rouzier R Verschueren PC van de Leij C Maas M Kraan MC Tak PP CCR5 blockade in rheumatoid arthritis: a randomised, double-blind, placebo-controlled clinical trial Ann Rheum Dis 2010 69 2013 2016 10.1136/ard.2010.131235 20693270

[B21] Rosario MC Jacqmin P Dorr P James I Jenkins TM Abel S Van Der Ryst E Population pharmacokinetic/pharmacodynamic analysis of CCR5 receptor occupancy by maraviroc in healthy subjects and HIV-positive patients Br J Clin Pharmacol 2008 65 Suppl 1 86 94 18333870 10.1111/j.1365-2125.2008.03140.x PMC2311409

[B22] Leech M Hutchinson P Holdsworth SR Morand EF Endogenous glucocorticoids modulate neutrophil migration and synovial P-selectin but not neutrophil phagocytic or oxidative function in experimental arthritis Clin Exp Immunol 1998 112 383 388 10.1046/j.1365-2249.1998.00601.x 9649205 PMC1905001

[B23] Goulding NJ Euzger HS Butt SK Perretti M Novel pathways for glucocorticoid effects on neutrophils in chronic inflammation Inflamm Res 1998 47 Suppl 3 S158 S165 9831319 10.1007/s000110050310

[B24] US Food and Drug Adminstration SELZENTRY (maraviroc) tablets Highlights of Prescribing Information 2009 http://www accessdata fda gov/drugsatfda_docs/label/2007/022128lbl pdf

[B25] Abel S van der Ryst E Rosario MC Ridgway CE Medhurst CG Taylor-Worth RJ Muirhead GJ Assessment of the pharmacokinetics, safety and tolerability of maraviroc, a novel CCR5 antagonist, in healthy volunteers Br J Clin Pharmacol 2008 65 Suppl 1 5 18 18333861 10.1111/j.1365-2125.2008.03130.x PMC2311414

[B26] Yost R Pasquale TR Sahloff EG Maraviroc: a coreceptor CCR5 antagonist for management of HIV infection Am J Health Syst Pharm 2009 66 715 726 10.2146/ajhp080206 19336831

[B27] Gulick RM Lalezari J Goodrich J Clumeck N DeJesus E Horban A Nadler J Clotet B Karlsson A Wohlfeiler M Montana JB McHale M Sullivan J Ridgway C Felstead S Dunne MW van der Ryst E Mayer H the MOTIVATE Study Teams Maraviroc for previously treated patients with R5 HIV-1 infection N Engl J Med 2008 359 1429 1441 10.1056/NEJMoa0803152 18832244 PMC3078519

[B28] Emmelkamp JM Rockstroh JK CCR5 antagonists: comparison of efficacy, side effects, pharmacokinetics and interactions--review of the literature Eur J Med Res 2007 12 409 417 17933722

[B29] Ryan CT Trials of aplaviroc halted in treatment-naive patients AIDS Clin Care 2005 17 107 108 16323301

[B30] Nichols WG Steel HM Bonny T Adkison K Curtis L Millard J Kabeya K Clumeck N Hepatotoxicity observed in clinical trials of aplaviroc (GW873140) Antimicrob Agents Chemother 2008 52 858 865 10.1128/AAC.00821-07 18070967 PMC2258506

[B31] Ndegwa S Maraviroc (Celsentri) for multidrug-resistant human immunodeficiency virus (HIV)-1 Issues Emerg Health Technol 2007 110 1 8 18080399

